# Risk behaviours among HIV positive injecting drug users in Myanmar: a case control study

**DOI:** 10.1186/1477-7517-7-12

**Published:** 2010-06-02

**Authors:** Lin A Swe, Kay K Nyo, AK Rashid

**Affiliations:** 1 Beneficial Partner Group, Myanmar. 30A 2 Inya Road. Kamayut Township, Yangon. Myanmar; 2 Department of Community Medicine, Faculty of Medicine, AIMST University, Jalan Bedong - Semeling, 08100 Bedong, Kedah Darul Aman, Malaysia; 3 Department of Public Health Medicine, Penang Medical College, 4 Sepoy Lines, 10450 Georgetown, Penang, Malaysia

## Abstract

**Background:**

The severity of HIV/AIDS pandemic linked to injecting drug use is one of the most worrying medical and social problems throughout the world in recent years. Myanmar has one of the highest prevalence rates of HIV among the IDUs in the region.

**Aim:**

The objective of the study was to determine the risk behaviours among HIV positive injecting drug users in Myanmar.

**Methods:**

A non matched case control study was conducted among 217 respondents registered with a non governmental organization's harm reduction center. 78 HIV positive IDUs were used as cases and 139 non HIV positive IDUs as controls. The study was conducted between April-May 2009. Data was analysed using SPSS version 15 and the study was ethically conducted.

**Results:**

Factors like age, marital status, age first used drugs, drug use expenditure, reason for drug use, age first used injection were found to be significant. Other risk factors found significantly associated with HIV among IDU were education (OR 2.3), location of respondent (OR 2.4) type of syringe first used (OR 5.1), sharing syringe at the first injection (OR 4.5) and failure of drug detoxification programme (OR 4.9). More HIV positive IDUs were returning used syringes in the centre (OR 3.3).

**Conclusions:**

Prudent measures such as access to sterile syringes and continuous health education programmes among IDUs and their sexual partners are required to reduce high risk behaviours of IDUs in Myanmar.

## Background

The severity of HIV/AIDS pandemic linked to injecting drug use is one of the most worrying medical and social problems throughout the world in recent years. Asia is by no means immune to this phenomenon where HIV is spreading early and rapidly among this group of people. In parts of northern Myanmar and several urban areas of Thailand, the prevalence of HIV infection up to levels of 40 to 80 percent has been recorded among injecting drug users (IDU) [1]. Although the prevalence of HIV levels are still very low in the Asia-Pacific region, the potential risk of spread of HIV infection among injecting drug users and the risk of spread of infection from them to others is worrisome.

IDUs form one of the major risk groups for HIV transmission in Myanmar. Myanmar has one of the highest prevalence rates of HIV among the IDUs in the region with 37.5% of the IDU population infected with HIV [2]. In Myanmar, people who are addicted to illicit drugs are required by law to register at the government drug treatment centres. Records show that 66,838 drug users were registered as of June 2003, of which 20% were injecting drugs. These IDU's were particularly vulnerable to HIV [3]. The actual numbers of drug users in Myanmar are still unknown as it is expected that most drug users do not register. The population size of IDUs in Myanmar generated during the estimation workshop held in Myanmar in 2004 ranged from 12,000-60,000. Other sources have quoted higher ranges from 150,000 to 250,000 [4] and 90,000 to 300,000 [4].

The objective of the study was to determine the risk behaviours among HIV positive injecting drug users in Myanmar.

## Methods

### Background place of study

The study was conducted in Shan state, near the Myanmar and Thailand border which has a high prevalence of IDU's in Myanmar. The centre where the programme is run has 22 staffs including medical doctors, nurses, counsellors, administrative staffs and outreach workers. The centre helps to implement harm reduction programmes in this part of Myanmar. This centre has collaborations with other Non Governmental Organizations (NGO) and local health authorities to prevent HIV transmission in the area. Besides syringe exchange, primary health care, referral of necessary cases, free condoms, treatment of sexually transmitted infections (STI), abscess dressing, hair dressing, bathing facilities, food supplies, socioeconomic support and income generation supports are provided by the center. The center also provides regular counseling and health education to IDUs. Outreach workers from the centre regularly go to the field to encourage drug addicts to utilize the services of the center, provide health education, exchange needle and syringes and search for new IDUs. Screening for HIV is provided free of cost through the referral network with the National AIDS Programme.

### Study design

A non matched case-control study design (the controls were not matched with the cases) was chosen to achieve the objective of the study. The study was conducted from January to March 2009 with the assistance of other NGOs operating in this area such as MSI and TOP.

### Sampling

The participants were recruited from among those attending the harm reduction programme run by a non governmental organization in Shan State. All IDUs who were registered at this centre were eligible to participate. Respondents who reported injecting drugs from six months to the time of the study were designated as "current IDUs".

HIV positive IDU's were recruited as cases and those IDUs who were not diagnosed as HIV were recruited as controls. Recruitment of the respondents was done with the help of the NGO which helped select respondents who attended the centre. The potential respondents were informed of the study via announcements made at the centre. The cases were defined as consenting IDU, aged 15 and above, diagnosed as HIV positive when screened using a HIV rapid test and confirmed by the second HIV rapid test (UNIGOLD). The controls were defined as consenting IDU, age 15 and above, diagnosed as HIV negative by HIV rapid test and those from the same community as the cases. The inclusion criteria included those who consented to participate and the exclusion criteria included those who refused to participate and were below the age of 15 years old.

### Tools

A quantitative questionnaire was developed and field tested prior to the actual study. The questionnaire had questions on demography, drug use history, daily drug expenditure, reuse and sharing of syringes, detoxification history and awareness about HIV. Interviews were conducted by volunteers who were expert in local languages. They were trained for three days and two days of field practice was conducted for them. The interviews were conducted in private settings.

### Laboratory Methods

The blood samples were taken from respondents after the interview and sent to site laboratories of the National AIDS Programme STI/AIDS Teams to perform HIV antibody tests. At the site laboratory, serum specimens were screened using a HIV rapid test kit (mostly Determine) and the reactive specimens were further confirmed by a second HIV rapid test kit (UNIGOLD) according to WHO testing strategy II.

### Ethics

This study was ethically conducted. A verbal consent was taken from each respondent before starting the interview and collecting the blood sample. The confidentiality of these patients was totally assured.

### Research Analysis

Data analysis was done using SPSS version 13.0. Descriptive statistics and cross tabulation were done. Chi square test was applied and the odds ratio was calculated.

## Results

All of the 217 registered injecting drug users responded to the study, giving the response rate as 100%. Seventy eight respondents were HIV positive and 139 were not. As shown in table 1, there were 211 male registered IDU's compared to 11 female registered IDUs. The age of the participants ranged from 18 to 54 years old with the mean age of 32.8 years. There were more HIV positive IDUs among those within the age group of 35 years and below (p = 0.02). Among the HIV positive IDUs there were more married respondents followed by singles and divorcees as compared with non HIV IDUs where most of the respondents were single followed by married and divorcees (p = 0.02). There is a two fold greater odds of having HIV when the IDU is illiterate. Majority of those with HIV positive IDUs were Shan followed by other races and Myanmar compared to non HIV IDUs where the majority were Myanmar followed by other races and Shans (p = 0.02). There was no significant difference in the religion among the HIV positive and non HIV IDUs. There was an almost three fold greater odds of having HIV when the IDU was from the rural area.

**Table 1:** Demographics

Variable		HIV N (%)		Chi squared	P value	Odds ratio	95% CI
		Yes	No				
**Sex**	Male	75 (96.1%)	136 (97.8%)				
	Female	3 (3.9%)	3 (2.2%)				
							
**Age**	<=35	42 (53.9%)	96 (69.1%)	4.99	0.03		
	>=36	36 (46.1%)	43 (30.9%)				
							
**Marital**	Single	24 (30.8%)	69 (49.6%)	9.00	0.03		
	Married	37 (47.4%)	55 (39.6%)				
	Divorce	15 (19.2%)	13 (9.5%)				
	Widow	2 (2.6%)	2 (1.4%)				
							
**Education**	Illiterate	18 (23.1%)	16 (11.5%)	5.06	0.02	2.31	1.09;4.83
	Literate	60 (76.9%)	123 (88.5%)				
							
**Race**	Myanmar	19 (24.4%)	57 (41.1%)	8.14	0.02		
	Shan	30 (38.4%)	32 (23.0%)				
	Other	29 (37.2%)	50 (35.9%)				
							
**Religion**	Buddhist	62 (79.5%)	110 (79.1%)				
	Christian	11 (14.1%)	15 (10.8%)				
	Muslim	5 (6.4%)	12 (8.6%)				
	Spiritual	0 (0%)	2 (1.4%)				
							
**Location**	Rural	51 (65.4%)	61 (43.9%)	9.25	0.00	2.42	1.36;4.29
	Urban	27 (34.6%)	78 (56.1%)				

### Patterns of drug usage among Injecting Drug Users (table 2)

**Table 2:** Patterns of Drug Usage among IDUs

Variable		HIV n (%)		Chi squared	p value	Odds ratio	95% CI
		Yes	No				
**First drug use experience**	Friends	62 (79.5%)	116 (83.5%)				
	Family	1 (1.3%)	0 (0%)				
	Sex worker	2 (2.6%)	0 (0%)				
	Other	13 (16.7%)	23 (16.5%)				
							
**First drug used #**	Opium	28 (31.5%)	32 (19.3%)				
	Heroin	46 (51.7%)	90 (54.2%)				
	Cough mixture	4 (4.5%)	6 (3.6%)				
	Diazepam	6 (6.7%)	11 (6.6%)				
	Marijuana	5 (5.6%)	27 (16.3%)				
							
**Present Drugs used #**	Opium	9 (9.8%)	9 (5.2%)				
	Heroin	71 (77.2%)	131 (76.2%)				
	Cough mixture	4 (4.4%)	2 (1.2%)				
	Diazepam	3 (3.3%)	15 (8.7%)				
	Marijuana	5 (5.4%)	15 (8.7%)				
							
**Age first used drugs**	<=21	45 (57.7%)	97 (69.8%)	3.23	0.05		
	=>22	33 (42.3%)	42 (30.2%)				
							
**Drug use frequency per day**	<=2	13 (16.7%)	26 (18.7%)				
	2-4	62 (79.5%)	106 (76.3%)				
	>=5	3 (3.9%)	7 (5.0%)				
							
**Drug use expenditure**	<=3000	38 (55.9%)	56 (40.3%)	3.55	0.05		
	3001-8000	20 (29.4%)	49 (35.3%)				
	>=8001	10 (14.8%)	34 (24.5%)				
							
**Reason for drug use**	Like	9 (11.5%)	24 (17.3%)	4.46	0.04		
	Experiment	21 (26.9%)	49 (35.3%)				
	Relaxation	3 (3.8%)	4 (2.9%)				
	Peer press	35 (44.9%)	49 (35.3%)				
	Upset	5 (6.4%)	11 (7.9%)				
	Other	5 (6.4%)	2 (1.4%)				

# Multiple response

For most of the responders, the first experience in using drugs was with friends. Heroin was the most common drug used for the first time and it was also the most common drug being used at the time of the study. The age range the responders first started using drugs was from 13 to 44, and the mean age was 20. The most common age for the first drug use was when they were 21 years old and below (p = 0.05). Most of the IDUs use drugs 2-4 times per day. The expenditure incurred for drug per day ranged from 88 to 70000 Myanmar Kyats (1000 Kyats = 1 US$) with the mean of 6591 (Kyats). Most of the IDUs spent less than 3001 Kyats. There were more HIV positive IDUs who spent less than 3001 Kyats but there were more non HIV IDUs who spent more than 3000 Kyats (p = 0.05). The most common reason for drug use was peer pressure and experimentation (p = 0.00).

### Trend of injecting drug (table 3)

**Table 3:** Trend of Injecting drugs

Variable		HIV		Chi squared	p value	Odds ratio	95% CI
		Yes	No				
**Method of Drug intake #**	Injection	78 (74.3%)	139 (46.6%)				
	Inhalation	15 (14.3%)	31 (10.4%)				
	Sniffing	4 (3.8%)	6 (2.0%)				
	Oral	8 (7.6%)	22 (7.4%)				
	Other	0 (0%)	100 (33.6%)				
							
**Age first IV**	<=21	23 (29.5%)	65 (46.8%)	6.189	0.02		
	=>22	55 (70.5%)	74 (53.2%)				
							
**IV location #**	Arm	41 (34.8%)	53 (31.55%)				
	Forearm	45 (38.1%)	96 (57.1%)				
	Finger	1 (0.9%)	1 (0.6%)				
	Thigh	8 (6.8%)	1 (0.6%)				
	Calf	5 (4.2%)	1 (0.6%)				
	Hand	15 (12.7%)	15 (8.9%)				
	Other	3 (2.5%)	1 (0.6%)				
							
**Type of syringe first use**	Used	44 (56.4%)	28 (20.1%)	26.64	0.00	5.13	2.79;9.44
	Disposable	34 (43.6%)	111 (79.9%)				
							
**Type of syringe used now**	Used	5 (6.4%)	3 (2.2%)				
	Disposable	73 (93.6%)	136 (97.8%)				
							
**Sharing Syringe at first injection**	Yes	47 (60.3%)	35 (25.2%)	26.15	0.00	4.50	2.49;8.16
	No	31 (39.7%)	104 (74.8%)				
							
**Sharing syringe now**	Yes	5 (6.4%)	7 (5.0%)				
	No	73 (93.6%)	132 (95%)				

# Multiple response

Injecting was the most common method of drug abuse. More HIV positive respondents started injecting when they were 22 years and above (p = 0.02). The common injecting sites were forearm, arm and hand. Those using used syringes were five fold greater risk of getting HIV as compared to those using disposable syringes for the first time. Those who share syringes were almost five times more likely to developed HIV compared with those who do not share syringes for the first time. The usage of used syringes as well as sharing syringes had reduced at the time the study was conducted.

### Knowledge and practice of prevention programmes (table 4)

**Table 4:** Knowledge and practice of prevention programmes

Variable		HIV		Chi squared	p	Odds ratio	95% CI
		Yes	No				
**Knowledge of Prevention programmes**	Yes	77 (98.7%)	137 (98.56%)				
	No	1 (1.3%)	2 (1.4%)				
							
**Easy to get syringe**	Yes	76 (97.4%)	136 (97.8%)				
	No	2 (2.6%)	3 (2.2%)				
							
**Will use more drugs with free syringes**	Yes	7 (9.0%)	15 (10.8%)				
	No	71 (91.0%)	124 (89.2%)				
							
**Returning the used syringe**	Yes	67 (85.9%)	90 (64.8%)	11.17	0.00	3.32	1.01;6.86
	No	11 (14.1%)	49 (35.3%)				
							
**Had Drug treatment**	Yes	73 (93.6%)	104 (74.8%)	11.19	0.00	4.91	1.84;13.14
	No	5 (6.4%)	35 (25.2%)				

Most of the respondents know about the Needle and Syringe Exchange Programme and they find it easy to get the syringes from the programme and most claim that they would not be inclined to use more drugs if they are given free syringes. HIV positive IDUs were three times more likely to return used syringes. Those with history of having unsuccessfully gone through drug treatment were almost five times more likely to be HIV positive.

## Discussion

**Sharing contaminated injecting equipment **is the primary mode of HIV transmission in many countries throughout Europe and Asia and it is now a major risk factor for the AIDS pandemic [5]. In this study we found that those who utilized used syringes for the first time were five times more likely be HIV positive and similarly those who shared syringe for the first time were five times more likely to be HIV positive. Evidence from other studies has shown the prevalence of HIV was highest among IDUs who reported daily injection and sharing syringes consistently [6].

This study also found that responders who were illiterate and those who lived in rural areas were twice likely to be HIV positive and most of the HIV positive responders were of the minority race. Similar findings were found in studies conducted elsewhere which found a higher prevalence of HIV among those with low education levels [7] and among the aborigines who lived in rural areas [8]. **A**mong the minority population in Myanmar most of them live in rural areas which have poor access to education system and harm reduction programmes provided by the government and NGO's. Report from the National AIDS programme showed a higher HIV prevalence among ethnic minorities. This could probably be because of the easy access to heroin in and around the permeable borders which has resulted in an increase in the injection mode of heroin or raw opium use especially among the ethnic minority populations [9]. As a result of increased use of injection mode of drug abuse there has been a rapid increase in the rate of HIV transmission among drug users in the border areas and northeast parts of Myanmar.

In this study most HIV positive responders were 35 years and below and most were married. A study conducted by Celentano [10] showed that those below the age of 39 years old had more than twice the incidence of HIV as did those aged 40 years and above [10]. Similarly, Myanmar HIV Sero-sentinel Survey conducted in 2008 reported that majority of HIV positive IDUs in Myanmar were 35 years and below [11]. A study by Jia [12] revealed that married IDUs have a greater risk of getting HIV than single or divorced IDUs.

This study also showed that HIV positive IDUs were more likely to return used syringes and those with a history of unsuccessful drug treatment were more likely to be HIV positive. There is substantial evidence that syringe exchange programs are effective in preventing HIV risk behaviour and HIV sero-conversion among IDUs [13-15]. The reduction of the HIV incidence and prevalence among the IDUs has successfully been achieved by the change of the high risk behaviours as a result of harm reduction programmes [15-17].

### Limitations

There are a number of limitations in this study. The total number of study participants was less than optimal and the study is limited by its non matched case control design.

## Conclusions

Illiterates and the minority IDU's are at a higher risk of being HIV positive. Health intervention programmes should focus on these vulnerable groups with the emphasis on the harmful effects of sharing needles.

## Competing interests

The authors declare that they have no competing interests.

## Authors' contributions

LAS performed the field work and the data collection, whereas LAS and KKN planned the write up of the article. Data was analysed by ARK.

The authors have read and approved the manuscript.
